# Plasma small extracellular vesicles from dogs affected by cutaneous mast cell tumors deliver high levels of miR-21-5p

**DOI:** 10.3389/fvets.2022.1083174

**Published:** 2023-01-10

**Authors:** Clarissa Zamboni, Valentina Zamarian, Damiano Stefanello, Roberta Ferrari, Luigi Auletta, Samantha Milanesi, Samuele Mauri, Valeria Grieco, Fabrizio Ceciliani, Cristina Lecchi

**Affiliations:** ^1^Dipartimento di Medicina Veterinaria e Scienze Animali, Università degli Studi di Milano, Milan, Italy; ^2^Diabetes Research Institute, IRCCS San Raffaele Hospital, Milan, Italy; ^3^Leukocytes Biology Group, IRCCS Humanitas Clinical and Research Center, Milan, Italy; ^4^Department of Medical Biotechnologies and Translational Medicine, Università degli Studi di Milano, Milan, Italy

**Keywords:** dog, mast cell tumor, exosome (vesicle), miR-21-5p, size exclusion chromatography

## Abstract

Small extracellular vesicles (sEV) are a class of extracellular vesicles (30–150 nm), delivering molecules including proteins, metabolites, and microRNAs (miRNAs), involved in physiological intercellular crosstalk and disease pathogenesis. The present pilot study aims are (I) to develop an easy and fast protocol for the isolation of sEV from plasma of mast cell tumor (MCT)-affected dogs; (II) to evaluate if miR-21-5p (sEV-miR-21-5p), a miRNA overexpressed by MCT, is associated with sEV. Seventeen dogs have been enrolled in the study: 4 healthy and 13 (6 with and 7 without nodal metastasis) MCT-affected dogs. sEV were isolated using size exclusion chromatography (SEC) (IZON column 35nm) and were characterized by Western blot, Nanoparticle tracking analysis, and transmission electron microscopy. sEV-miR-21-5p was quantified using digital PCR. sEV expressed the specific markers CD9 and TSG101, and a marker of mast cell tryptase. The sEV mean concentration and size were 2.68E + 10 particles/ml, and 99.6 nm, 2.89E + 10 particles/ml and 101.7 nm, and 3.21E + 10 particles/ml and 124 nm in non-metastatic, nodal metastatic, and healthy samples, respectively. The comparative analysis demonstrated that the level of sEV-miR-21-5p was significantly higher in dogs with nodal metastasis compared to healthy (*P* = 0.038) and without nodal metastasis samples (*P* = 0.007). In conclusion, the present work demonstrated that a pure population of sEV can be isolated from the plasma of MCT-affected dogs using the SEC approach and that the level of sEV-miR-21-5p is higher in nodal metastatic MCT-affected dogs compared with healthy and MCT-affected dogs without nodal involvement.

## Introduction

Canine mast cell tumor (MCT) is one of the most frequent malignant tumors in dogs representing around 17.8% of cutaneous neoplasms (1, 2). Although adequate local therapy alone is sufficient in the treatment of most cases, there is a subset of dogs that develops recurrence and metastatic disease (3–5). The histological grade of the primary mass is the most commonly used parameter for predicting the biological behavior of the tumor (6, 7) in association with staging status. In particular, a histological classification system that standardizes the evaluation of lymph node MCT-metastasis has been described (8). Lymph nodes of MCT-affected dogs are classified into four different classes with a correlation to the clinical outcome (8) but the specific assessment of the metastatic potential or disease to properly calibrate therapy and predict the prognosis is still challenging (9). Furthermore, the timing of follow-up or restaging in treated patients with the predictable minimal residual disease is anecdotal and may benefit from future improvements to control tumor recurrence.

Extracellular vesicles (EVs) are particles released by all cell types in normal and pathological conditions (10) and are present in different body fluids, including plasma, urine, milk, sweat, tears, saliva, and cerebrospinal fluid (11). They are involved in cell-to-cell communication by carrying small and long RNAs, proteins, lipids, metabolites, and DNA fragments, which can induce phenotypic reprogramming of recipient cells (12, 13). EVs are classified into three main subtypes identified by the biogenesis pathway: Small extracellular vesicles (sEV) (30–120 nm), microvesicles (100–1,000 nm), and apoptotic bodies (800 to 5,000 nm) (10). It is reported that tumor cells communicate with each other and with surrounding cells, like immune cells, fibroblasts, and endothelial cells, through EVs, which thus play a crucial role in all steps of tumor progression (14). Tumor EVs can reprogram a recipient cell inducing a tumor-promoting phenotype and modifying the microenvironment cells to support tumor growth, survival, local invasion, metastasis, and also to sustain angiogenesis, resistance to cell death, evasion from the immune response, reprogramming of cellular metabolism, and the drug resistance (15). sEV deliver molecules that reflect the parental features and molecules specific to the disorders and diseases, including cancers, pointing out their potential as targets for liquid biopsies for early diagnosis, prognosis, cancer staging and patient follow-up (16).

MicroRNAs (miRNAs) are small non-coding RNAs involved in cellular processes epigenetically modulating the mRNA transcription in different pathophysiological disorders (17, 18). miRNAs could circulate free in the plasma or can be delivered by EV and the sEV cargoes have engendered huge interest for clinical application as diagnostic biomarkers and therapeutic cargo carriers (19, 20). Deregulation in miRNAs expression correlates with various cancers by acting either as tumor suppressors or oncogenes. Their potential as predictive biomarkers to diagnose and predict cancer stages in human and veterinary medicine has been reported (21–25).

The miRNAs profile of canine MCT has been previously characterized in formalin-fixed paraffin-embedded samples pointing out that a panel of three miRNAs, miR-21-5p, miR-379, and miR-885, have a good efficiency in discriminating healthy and MCT affected dogs, and MCT affected dogs with and without nodal metastasis, suggesting an epigenetic role in tumor progression (26). Recently, miR-21-5p has been identified as a potential biomarker in the saliva of MCT-affected dogs (27). No data on plasma circulating miR-21-5p in MCT-affected dogs have been reported so far. Several studies have been conducted in veterinary oncology to investigate the sEV cargo and their role in tumors (28–32), but only one study investigated the concentration of EVs in the blood of MCT-affected dogs with different histological grades (33). Based on the above considerations, this pilot study aimed at evaluating the expression of miR-21-5p in the plasma of dogs affected by cutaneous MCT to confirm the ability of miR-21-5p to be associated with sEV and investigate whether the stage and consequently the prognosis of the disease are correlated to the sEV-mi-R-21-5p level supporting its potential as a biomarker for canine MCT.

## Materials and methods

### Samples selection

Seventeen plasma samples were collected from 4 healthy and 13 cutaneous MCT-affected (7 non-metastatic and 6 with nodal metastasis) client-owned dogs (Supplementary Table 1) referred to the University Veterinary Teaching Hospital of the University of Milan. Owners signed written consent for the procedure. All experimental procedures were reviewed and approved by the Ethics Committee of the University of Milano (approval number 118/19). Patients were recruited after written owner consent. All experiments were performed following the relevant guidelines and regulations.

In the MCT group, dogs with a cytological diagnosis of MCT, that were preoperatively staged negative for distant metastases (34) and underwent wide margins surgical excision of the MCT and biopsy of the sentinel lymph node(s) (SLN), without any medical treatment for at least 14 days before surgery, were included (35, 36). Histopathology was performed on both primary tumor and SLN samples as previously reported (27). The MCTs were grouped based on SLN involvement in two classes: non-metastatic/pre-metastatic (HN0-1 class) and early-metastatic/overt-metastatic (HN2-3 class) (8). In the case of a biopsy of more than one SLN in the same dog, the major class of metastasis was considered. For the healthy group, randomly selected dogs among those admitted for routine annual clinical examination and vaccination were included, without either MCT or any other oncological or systemic diseases, and in which clinicians decided to collect the peripheral blood sample for check-up analysis. Blood samples were collected immediately before surgery into Monovette EDTA tubes (Sarstedt Company, Nümbrecht, Germany) and centrifuged at 800 × g for 15 min. Plasma was stored at −80°C until use.

### Extracellular vesicles isolation

Extracellular Vesicles (EVs) were purified from 0.5 ml of plasma by Size Exclusion Chromatography (SEC) using the qEV original – 35 nm columns (IZON, Cat. qEVoriginal 35 nm). The sample was depleted of cell debris by a series of centrifugations at 1,000, 2,000, 3,000 x g for 15 min at 4°C. Samples were loaded into the qEV column and the EVs isolation was performed following the manufacturer's procedure. Briefly, after sample loading, the first 3.0 ml were discarded and the next three fractions (0.5 ml each) containing the EVs were collected. The column equilibration and the fractions' elution were carried out using sterile three times-filtered (0.22 μm) Ammonium Bicarbonate buffer 20 mM pH 7.5.

The protein concentration was assessed using the Pierce^TM^ BCA Protein Assay kit (Thermo Fisher Scientific, Cat. No. 23225) following the manufacturer's procedure. Blue Coomassie staining was performed to evaluate the depletion of plasmatic proteins. Four micrograms of total protein of each fraction and 1 μl of whole plasma were loaded on a 12% SDS-polyacrylamide gel and stained with Blue Coomassie. The bands were visualized after a destining step with a solution of 30% methanol and 10% acetic acid.

### Western blot analysis

Western blot analysis was performed to assess the presence of EV and mast cell markers in the purified samples. Four μg of total proteins were loaded. Following SDS PAGE, proteins were transferred to nitrocellulose membranes using Trans-Blot Turbo Midi 0.2 μm Nitrocellulose Transfer Packs (Biorad, Cat. No. 1704159) on the Trans-Blot Turbo Transfer System (Biorad). The membrane was blocked for 1 h with ROTI^®^Block 1X (Carlroth, Cat. No. A151.1), incubated with primary antibodies anti-CD9 (Biorad, MCA469GT, 1:1,500), or anti-mast cell tryptase (Dako, M7052, 1:1,000) overnight at 4°C and then with the secondary antibody polyclonal anti-mouse peroxidase (Dako, P0260, 1:2,000) for 1h at room temperature; or with the primary antibody anti-TSG101 (Abcam, ab225877, 1:2,000) overnight at 4°C and secondary antibody polyclonal anti-rabbit peroxidase (Vector, PI-1000, 1:3,000) for 1 h at room temperature. Immunoreactive bands were visualized by enhanced chemiluminescence (Millipore, Cat. No. WBKLS0050).

### Electron microscopy and nanoparticle tracking analysis

To assess the morphology, EV were visualized using negative staining by TEM. Few microliters of samples were absorbed on glow-discharged carbon-coated formvar copper grids contrasted with 2% uranyl acetate, air-dried, and observed in an FEI Talos 120 kV transmission electron microscope (FEI Company, Netherlands). Images of EV were acquired by a 4k × 4K Ceta CMOS camera.

The size and concentration of EVs were assessed using the NanoSight NS300 (Malvern Panalytical). The samples were diluted 50 or 100 times using fresh three-times filtered PBS (0.22 μm). Particles were visualized and analyzed by the NTA 3.3 Dev Build 3.3.301 software. The instrument set up was to operate at 22°C, syringe pump speed 30 AU, and for each sample were recorded 5 videos of 60 s each; results were the mean of the 5 measurements.

### EVs miRNA extraction and reverse transcription

Small RNAs were extracted using microRNA concentrator kit (A&A Biotechnology, Cat. No 035-25) following the manufacturers' instructions. Since the EV with the size ranged in the expected size of sEV were eluted in fractions 2 and 3, only these fractions were included in the analysis. The *Caenorhabditis elegans* miRNA cel-miR-39 (25 fmol final concentration) (Qiagen, Cat. No 219610) was selected as a synthetic spike-in control because of a lack of sequence homology to canine miRNAs. The quantifications of RNA were determined using a NanoDrop^TM^ Lite Spectrophotometer (Thermo Fisher Scientific).

Reverse transcription was performed on 16 ng of RNA of each sample using TaqMan Advanced miRNA cDNA Synthesis KIT (Cat. No A28007, Applied Biosystems), following the manufacturer's instructions.

To check extraction and retrotranscription reactions, the spike-in cel-miR-39 was quantified by qPCR following the MIQE Guidelines (37). The quantitative reaction was performed in duplicate with a reaction volume of 15 μl on CFX Connect Real-Time PCR Detection System (Biorad) using 7.5 μl of 2X TaqMan Fast Advanced Master Mix (Cat. No 4444557), 0.75 μl of 20x cel-miR-39 probe (assay ID 478293_mir), 1 μl of cDNA diluted in sterile water to reach the final volume. The thermal profile was 50°C for 2 min, 95°C for 3 min, and 40 cycles of 95°C for 15 s and 60°C for 40 s.

### Digital PCR (dPCR)

Digital PCR was carried out on the QuantStudio^TM^ 3D Digital PCR Instrument (Thermo Fisher Scientific). The reaction volume of 16 μl included 2 μl of cDNA, 8 μl of QuantStudio™ 3D Digital PCR Master Mix v2 (Thermo Fisher Scientific, Cat. No A26359), 0.8 μl of the rno-miR-21-5p (assay ID rno481342_mir) (20x), and 5.2 μl of nuclease-free water. Reaction tubes were gently vortexed and 15 μl of the product was loaded into the QuantStudio 3D Digital PCR Chip v2 (Thermo Fisher Scientific, Cat. No A26317). The thermal cycling profile was as follows: 96°C for 10 min, 40 cycles at 60°C for 80 s and 98°C for 30 s, and 60°C for 2 min. Quantification was performed using the QuantStudio 3D AnalysisSuite (Thermo Fisher Cloud) after setting the same threshold for all the samples. Data are presented as copy numbers/μl.

### Statistical analysis

XLStat software for Windows (Addinsoft, New York, USA) was used to perform the statistical analysis. The non-parametric Kruskal-Wallis and *post-hoc* Dunn test for multiple pairwise comparisons was applied as data were not normally distributed according to the Shapiro-Wilk test. Statistical significance was accepted at *P* ≤ 0.05. The area under the curve (AUC), confidence interval, sensitivity, and specificity were analyzed by a receiver operating characteristic (ROC) curve.

## Results

### EVs isolation and characterization

**Figure 1:**
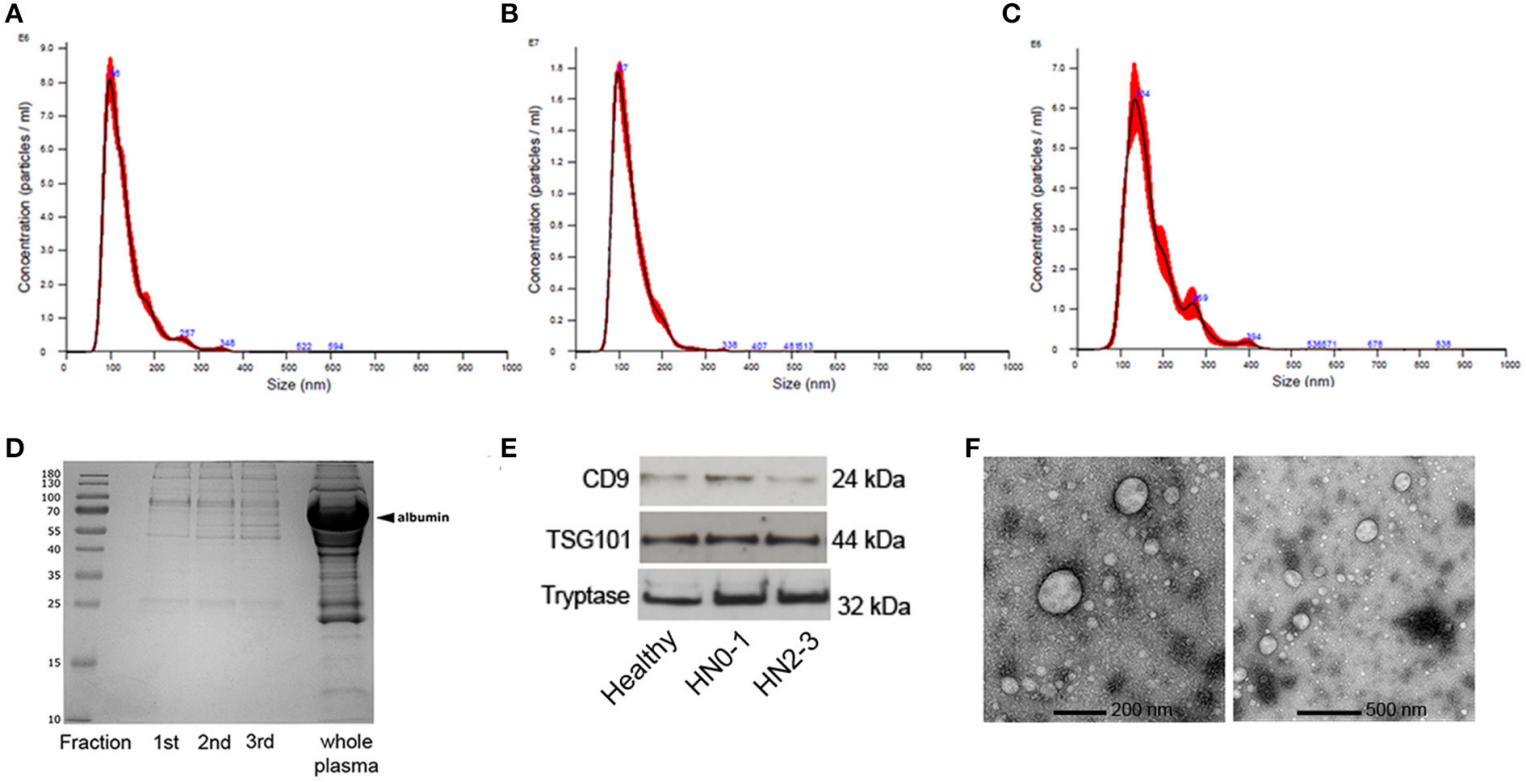
Characterization of extracellular vesicles (EVs) isolated by Size Exclusion Chromatography (SEC) from plasma of dogs. Nanoparticle tracking analysis of EVs from **(A)** non-metastatic/pre-metastatic (HN0-1) MCT. **(B)** Early-metastatic/overt-metastatic (HN2-3) MCT and **(C)** healthy sample. **(D)** Blue Coomassie staining of the three SEC fractions and the whole plasma. **(E)** sEV were loaded onto separate lanes, subjected to SDS PAGE, and transferred to nitrocellulose membranes; the immunoreactivity was detected with mouse anti-human CD9, rabbit anti-human TSG101, and mouse anti-human mast cell tryptase using ECL detection. **(F)** Transmission electron microscopy of the sEVpurified from plasma of dogs with MCT by SEC with a lower (200 nm) and a higher (500 nm) magnification.

The concentration and size of the EVs were assessed by Nanosight (Figures 1A–C). The median concentration of the EVs in non-metastatic/pre-metastatic (HN0-1) MCT samples was 2.69E+10 particles/ml (min = 1.37E+10 particles/ml; max = 3.98E+10 particles/ml), in early-metastatic/overt-metastatic (HN2-3) MCT samples was 2.03E+10particles/ml (min = 8.95E+09 particles/ml; max = 5.95E+10 particles/ml), and in healthy samples was 2.32E+10 particles/ml (min = 2.07E+10 particles/ml; max = 6.13E+10 particles/ml). Most of EVs were in the expected morphological sEV range, with a modal size of 99.6 nm (min = 66.6 nm; max = 124.5 nm) in non-metastatic/pre-metastatic (HN0-1) MCTs, 101.7 nm (min = 90 nm; max = 108.3 nm) in early-metastatic/overt-metastatic (HN2-3) and 124 nm (min = 110.4 nm; max = 143.8 nm) in healthy samples. No significant differences were detected between healthy, HN0-1, and HN2-3 classes in terms of vesicle concentration and size.

The protein concentration means were 266.7 μg/mL ± 125.3 μg/mL Standard Deviation (SD), 369,7 μg/mL ± 130.7 μg/mL SD, and 514.4 μg/mL ± 99 μg/mL SD in HN0-1, HN2-3 and healthy dogs. Comparing the protein profiles of fractions obtained by SEC isolation and whole plasma using blue Coomassie staining, the result highlighted that SEC depleted the most abundant plasmatic proteins (Figure 1D). The EVs markers protein CD9 and TSG101, and the mast cell marker tryptase were evaluated by immunoblot analysis; the bands with the expected Molecular Weight were visualized in all tested samples and the positive control (Figure 1E). Transmission electron microscopy showed that sEV exhibited the expected round vesicular membranes 30–150 nm in diameter and the ultrastructure of sEV-like vesicles (Figure 1F; Supplementary Figure 1).

### A high level of miR-21-5p is associated with sEV of metastatic MCT-affected dogs

Small RNA was isolated and reverse-transcripted cDNA was obtained from all samples included in the study. The synthetic spike cel-miR-39 was detected in all samples at the expected Cq (23 ≤ Cq ≤ 25) confirming the success of extraction and retrotranscription steps.

**Figure 2:**
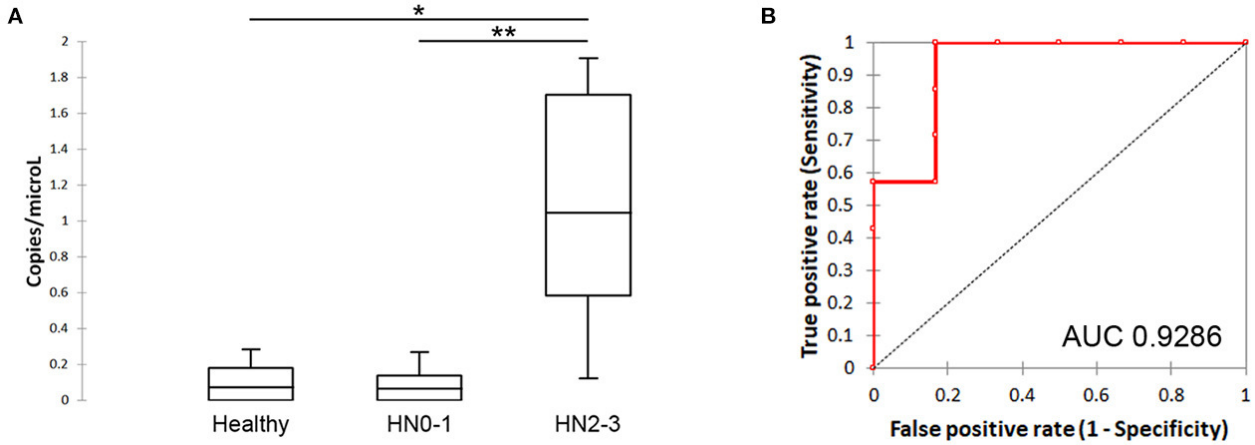
Quantification of sEV-miR-21-5p and evaluation of its ability to discriminate the metastatic patients. **(A)** Box plot of sEV-miR-21-5p in healthy and MCT-affected dogs without (HN0-1) or with (HN2-3) nodal metastasis. **(B)** Performance of sEV-miR-21-5p as a candidate biomarker for discriminating MCT-affected dogs without (HN0-1) or with (HN2-3) nodal metastasis. Receiver-operator characteristic (ROC) curve analysis. AUC, area under the curve.

The comparative analysis demonstrated that the level of sEV-miR-21-5p was significantly higher in nodal metastatic MCT-affected dogs (HN2/3; mean: 3.12 copies/μL; min:0.12 copies/μL; max: 14.15 copies/μL) compared to healthy (mean: 0.11 copies/μL; min:0 copies/μL; max: 0.28 copies/μL; *P* = 0.038) and non-metastatic (HN0/1) (mean: 0.09 copies/μL; min: 0 copies/μL; max: 0.27 copies/μL; *P* = 0.007) MCT derived plasma samples (Figure 2A). No statistical differences were found between healthy and non-metastatic MCT plasma samples.

The ability of sEV-miR-21-5p to discriminate the metastatic patients was defined as “diagnostic accuracy”. It was measured by the area under the curve (AUC). sEV-miR-21-5p proved to be efficient in discriminating between metastatic and non-metastatic dogs (AUC = 0.9286; 95% CI = 0.9286–0.9286) (Figure 2B). Setting the cut-off at 0.2697, the sensitivity was 100% and the specificity was 83.33%. sEV-miR-21-5p can efficiently discriminate between metastatic and healthy dogs (AUC = 0.9167; 95% CI = 0.9167–0.9167) (Supplementary Figure 2), and setting the cut-off at 0.2848 the sensitivity was 100% and the specificity was 80%.

## Discussion

Several studies in human medicine have tried to identify novel diagnostic and prognostic biomarkers for the management of cancer patients 11, (38–41). New kinds of biomarkers with performant sensitivity and specificity are required to assess the same in veterinary medicine, especially for high-incidence cancer diseases. To the best of our knowledge, this is the first report dealing with the plasma concentration of sEV-miR-21-5p in MCT-affected dogs.

sEV are continuously released and cumulated in biological fluids (11), acquiring fame as important targets for liquid biopsy. A protocol for the isolation, quantification, and characterization of plasmatic sEV of dogs with MCT, depleted from the most abundant plasmatic proteins, was assessed. Plasma EVs structures were observed under transmission electron microscopy, confirming the presence of particles in the range of 30–120 nm, which is consistent with sEV morphology and the integrity of their membrane (42, 43). The positivity in WB detection of sEV markers (CD9, TSG101) was in agreement with previous reports (44). Moreover, the positivity of samples to tryptase indicated that mast cells may secrete sEV into the bloodstream influencing target cell behavior. The EVs released specifically by human mast cells during systemic mastocytosis play a role in the disease influencing different organs (45). The development of a good and standardized protocol for the isolation of canine MCT-derived sEV could provide a step forward in disclosing the molecular strategy of MCT to communicate with cells far from its primary mass and prepare metastatic niches influencing the microenvironment.

MiRNA is one of the most recent functionally re-evaluated nucleic acids that could mostly circulate free or not randomly incorporated inside sEV (46), primarily in the case of pathological conditions (47, 48). The use of miRNAs as biological indicators especially for prognosis in cancer diseases is becoming more common in human medicine (47, 49). For the reported great stability and resistance of the sEV layer, sEV-miRNAs are described as even more purposing non-invasive cancer biomarkers in different biological fluids (50–52).

Circulating free-miRNAs have been investigated in a few studies in veterinary medicine. They were examined for parasitic diseases, cardiovascular injuries, neuropathies, endocrinopathies, drug interactions, and cancer (53–58). sEV-miRNA research in veterinary medicine is still in its first steps, with the majority of recently published studies about them focused on cancer diseases, such as canine oral melanoma, multicentric lymphoma, and lymphoid tumor cell lines (24, 30, 59). Studies dealing with sEV-miRNAs extracted from the plasma of dogs were very scarce (60, 61).

MiR-21-5p is one of the miRNA described as upregulated in canine MCT (26) and one of the first miRNA detected in humans as an oncomiR because of its overexpression associated with oncogenesis in different tumoral diseases (62–64). It is reported that its primary oncogenic mechanism involves the inhibition of *PTEN*, programmed cell death 4 (PDCD4), and other downstream target genes (65). Research in human medicine has validated the correlation of sEV-miR-21-5p expression with proliferation ability, invasion, and metastatic behavior (66, 67). To the best of our knowledge, no circulating sEV-miR21-5p expression and disease correlations have been explored in MCT in dogs. In the present study, plasma samples collected by healthy, non-metastatic (HN0/1) and nodal metastatic (HN2/3) MCT-affected dogs were collected and divided into 3 groups to quantify the concentration of circulating sEV-miR-21-5p. A multi-step approach, using EVs isolation protocol followed by the sample quality evaluation by RT-qPCR and the relief of absolute concentrations with chip-based digital PCR was assessed. Digital PCR is a recently developed technology that can be used to quantify absolute nucleic acid quantification and is increasingly accepted as the gold standard for biomarker evaluation because of its high precision and its lack of need for standardization by target molecules (68, 69).

The diagnostic accuracy of the data was excellent, with an AUC of 0.9286 for 100% sensitivity and 83% specificity. Our results provided for the first time the evidence that the expression level of circulating sEV-miR-21-5p changes in the plasma of dogs in different pathological stages (MCT with or without nodal metastasis), and that the level sEV-miR-21-5p was significantly higher in plasma collected from nodal metastatic MCT-affected dogs compared to healthy and early metastatic MCT-affected patients. These results suggest a possible role of sEV-miR-21-5p as an oncomiR in MCT, which contributes to the development of metastatic disease. Since the presence of nodal metastasis has been correlated with a more aggressive clinical behavior, sEV-miR-21-5p might be used as a biomarker for prognosis emission, or at least it could improve it. At the same time, considering the absence of prognostic factors guiding the surgeon in deciding whether to perform a lymph node extirpation or not (70), it might help in the identification of non-metastatic nodal MCT affected-dogs, avoiding unnecessary lymphadenectomy. Furthermore, the overexpression of sEV-miR-21-5p in the case of metastatic disease could improve the accuracy of clinical staging increasing the research for minimal residual disease. Further studies and larger samples of canine patients are necessary to explore and confirm these hypotheses.

The present study has limitations to be pointed out. The sample size is small and not balanced between healthy and MCT-affected dogs. However, even this pilot study performed on a limited number of dogs showed a statistical difference in the expression of sEV-miR-21-5p in metastatic MCT-affected dogs from non-metastatic MCT-affected and healthy ones suggesting the potential use of sEV-miR-21-5p as a candidate biomarker. Moreover, future studies including a higher number of patients could further improve the role as a suitable biomarker of sEV-miR-21-5p in veterinary oncology. The profiling of sEV cargo using OMICs technology may in-depth disclose the pathophysiological roles of these vesicles in tumor onset and development identifying also candidate new targets for the therapy.

In conclusion, the present work demonstrated that sEV can be isolated from the plasma of MCT-affected dogs with an easy and reproducible method and that the level of sEV-miR-21-5p is higher in nodal metastatic MCT-affected dogs compared with healthy and MCT-affected dogs without nodal involvement.

## Data availability statement

The original contributions presented in the study are included in the article/Supplementary material, further inquiries can be directed to the corresponding author.

## Ethics statement

The animal study was reviewed and approved by Animal Welfare Organization of the University of Milan. Written informed consent was obtained from the owners for the participation of their animals in this study.

## Author contributions

CL, CZ, and VZ designed the research, performed the statistical analysis, and wrote the paper. VZ, CZ, SMa, SMi, FC, and CL conducted the experiments, collected the data, and performed laboratory experiments. DS, RF, and LA enrolled patients and surgically removed the tumors. VG performed histologically classified and assessed grading of tumors. All authors have read and approved the final manuscript.
