# Supplementary material for: Plasma small extracellular vesicles from dogs affected by cutaneous mast cell tumors deliver high levels of miR-21-5p
Source: Front Vet Sci. 2023 Jan 10;9:1083174. doi: 10.3389/fvets.2022.1083174 (PMC9871458; doi:10.3389/fvets.2022.1083174)
Supplement: Supplementary file 1 [file Table_1.docx]

**Supplementary Table 1.** Clinical and histopathological data of dogs enrolled in the study.

| ID | Breed | Gender | Weight (kg) | Age (years) | Tumor location | Site | Grade |  | Lymph node* |
| --- | --- | --- | --- | --- | --- | --- | --- | --- | --- |
|  |  |  |  |  |  |  | Patnaik | Kiupel |  |
| 1 | Labrador | Male | 38 | 10 | Scrotum | Cutaneous | II | Low | HN0 |
| 2 | Dachshund | Female | 5 | 9 | Tail | Cutaneous | II | Low | HN0 |
| 3 | Mixed breed | Female | 34 | 10 | Forelimb | Cutaneous | II | Low | HN0 |
| 4 | Mixed breed | Female | 32 | 9 | Hindlimb | Cutaneous | II | Low | HN0 |
| 5 | Swiss mountain dog | Male | 38 | 6 | Forelimb | Cutaneous | I | Low | HN1 |
| 6 | Pug | Male | 9 | 7 | Forelimb | Cutaneous | II | Low | HN1 |
| 7 | Cocker spaniel | Male | 18 | - | Trunk | Cutaneous | II | Low | HN1 |
| 5 | Golden Retriever | Female | 26 | 7 | Forelimb | Cutaneous | II | Low | HN2 |
| 6 | Tosa Inu | Male | 55 | 4 | Foreskin | Cutaneous | II | Low | HN2 |
| 7 | Boxer | Male | 38 | 8 | Hindlimb | Cutaneous | II | Low | HN2 |
| 8 | Weimaraner | Male | 35 | 9 | Forelimb | Cutaneous | II | Low | HN2 |
| 9 | Boxer | Male | 36 | 8 | Hindlimb | Cutaneous | II | Low | HN2 |
| 10 | Golden Retriever | Female | 32 | 9 | Forelimb | Cutaneous | II | Low | HN2-3 |
| 11 | Italian spaniel | Female | 30 | 8 | - | - | - | - | Healthy |
| 12 | Pointer | Female | 23 | 9 | - | - | - | - | Healthy |
| 13 | Mixed breed | Male | 32 | 8.5 | - | - | - | - | Healthy |
| 14 | Labrador | Female | 35 | 6 | - | - | - | - | Healthy |

*classification system proposed by Weishaar and colleagues (2014). HN= histological node
