# Supplementary material for: Plasma small extracellular vesicles from dogs affected by cutaneous mast cell tumors deliver high levels of miR-21-5p
Source: Front Vet Sci. 2023 Jan 10;9:1083174. doi: 10.3389/fvets.2022.1083174 (PMC9871458; doi:10.3389/fvets.2022.1083174)
Supplement: Supplementary file 2 [file Data_Sheet_1.docx]

-
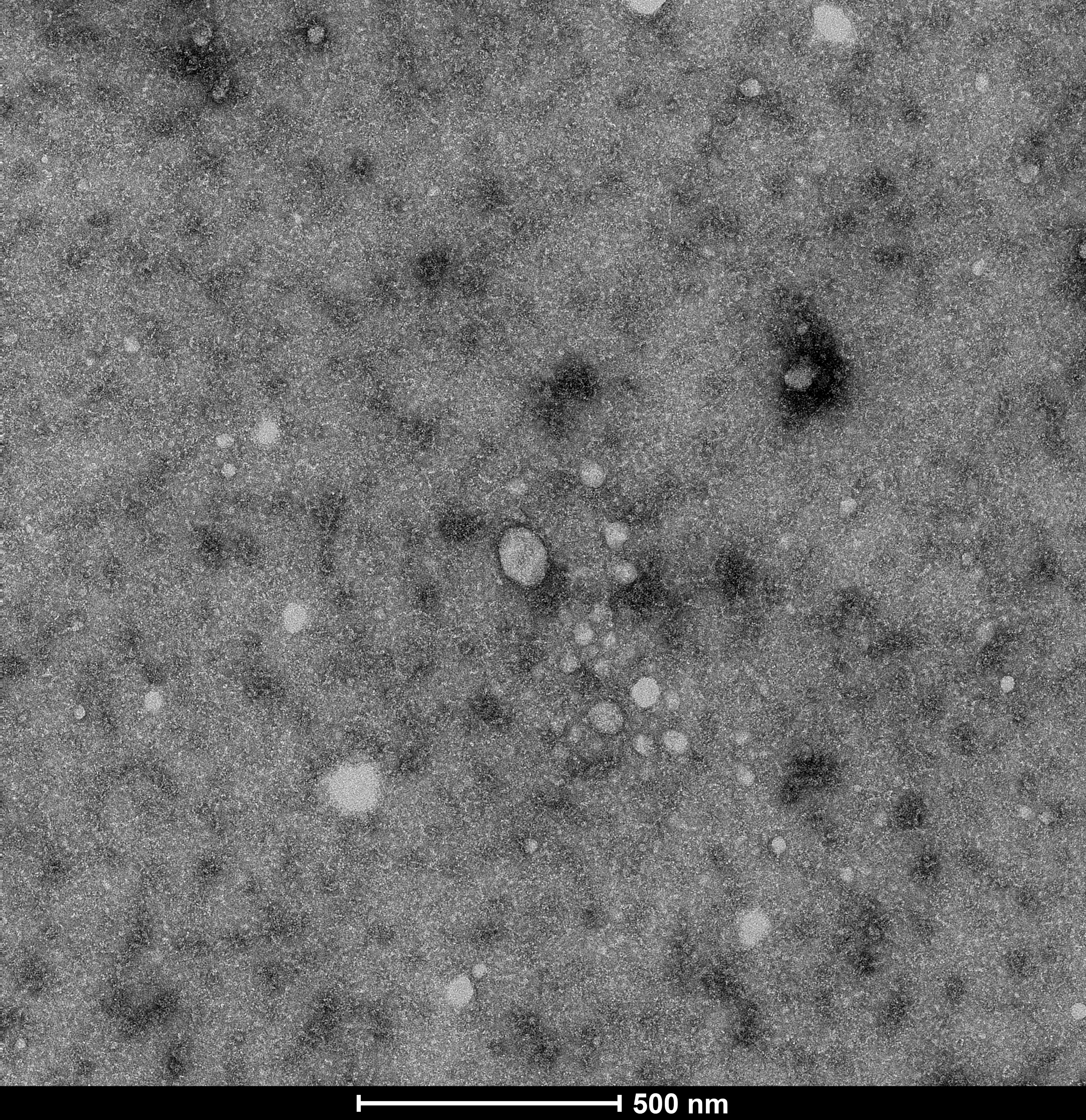

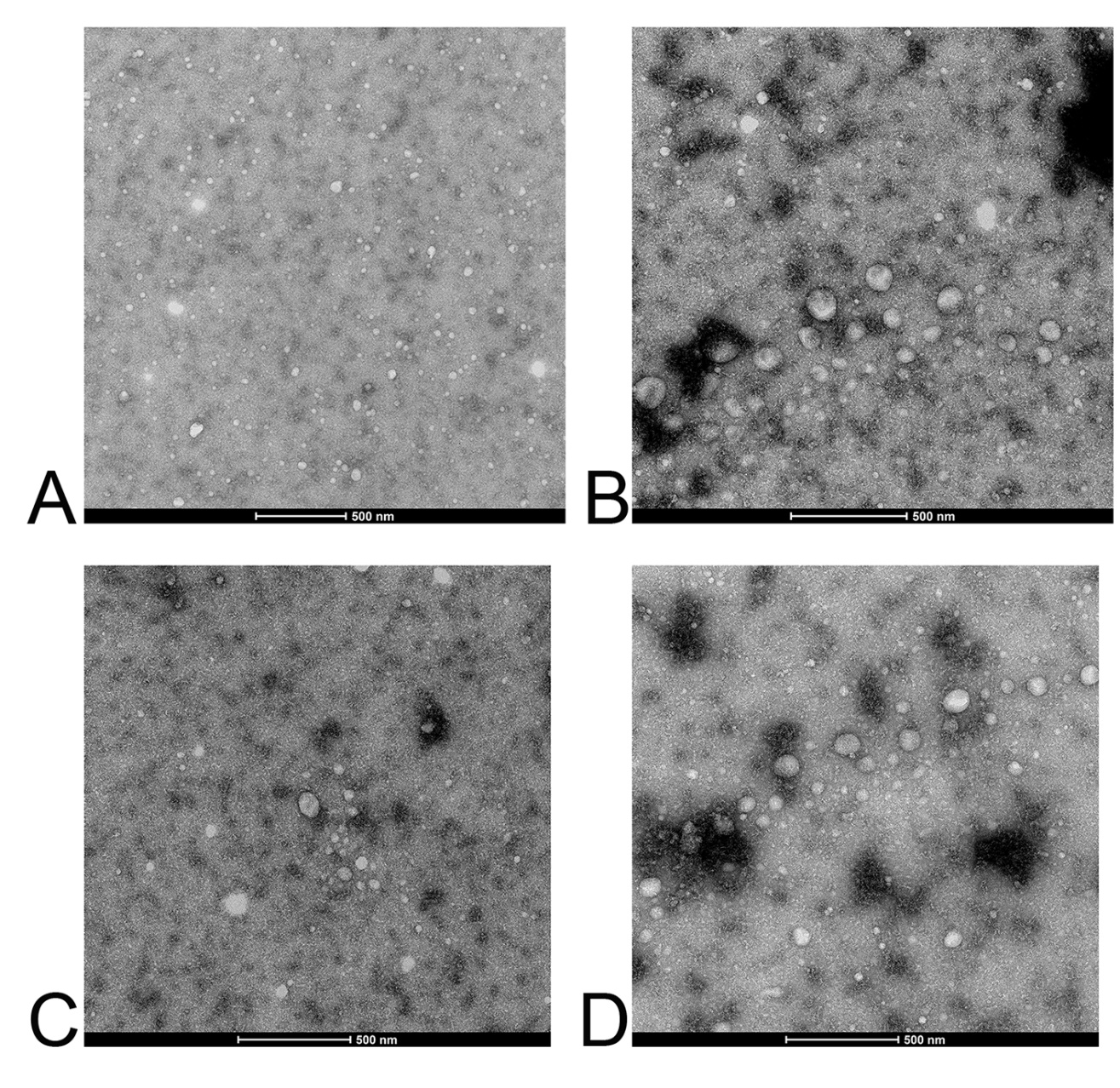


**Supplementary Figure 1.** transmission electron microscopy of the sEVpurified from plasma of dogs with MCT by SEC with a higher (500 nm) magnification.
