# Supplementary material for: Plasma small extracellular vesicles from dogs affected by cutaneous mast cell tumors deliver high levels of miR-21-5p
Source: Front Vet Sci. 2023 Jan 10;9:1083174. doi: 10.3389/fvets.2022.1083174 (PMC9871458; doi:10.3389/fvets.2022.1083174)
Supplement: Supplementary file 3 [file Data_Sheet_2.docx]

**
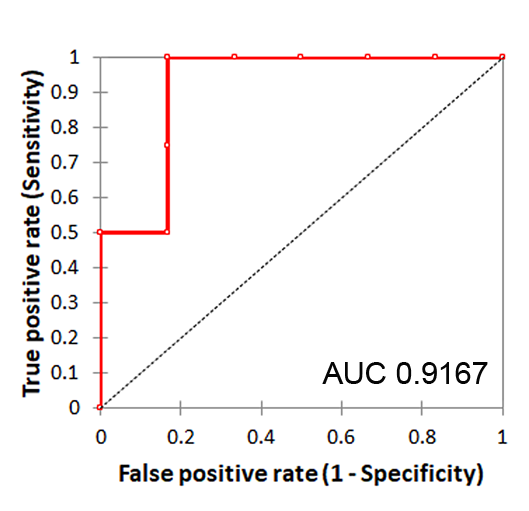
**

**Supplementary Figure 2.** Performance of exo-miR-21-5p as a candidate biomarker for discriminating healthy and MCT-affected dogs with nodal metastasis(HN2-3). Receiver-operator characteristic (ROC) curve analysis. AUC= area under the curve.
